# Supplementary figures and images for: Factors associated with unsuccessful tuberculosis treatment among homeless persons in Brazil: A retrospective cohort study from 2015 to 2020
Source: PLoS Negl Trop Dis. 2023 Oct 20;17(10):e0011685. doi: 10.1371/journal.pntd.0011685 (PMC10619819; doi:10.1371/journal.pntd.0011685)

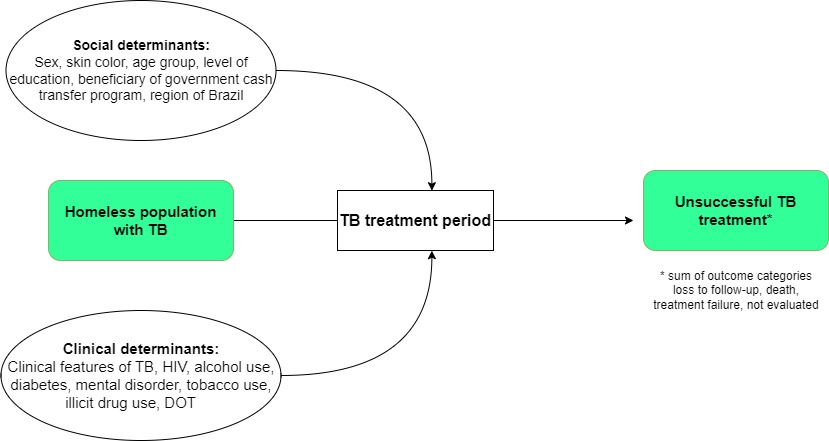

Supplement: S1 Fig — (TIF) [file pntd.0011685.s001.tif]

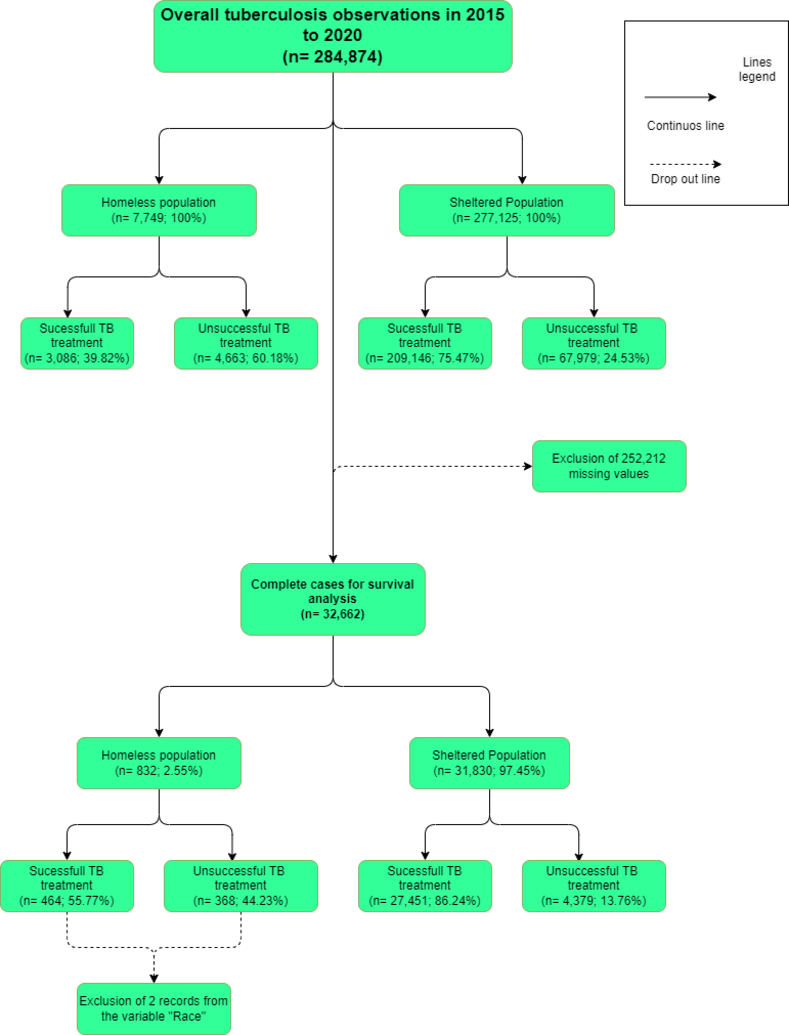

Supplement: S2 Fig — (Data source: SINAN—tuberculosis updated in August 2021). (TIF) [file pntd.0011685.s002.tif]

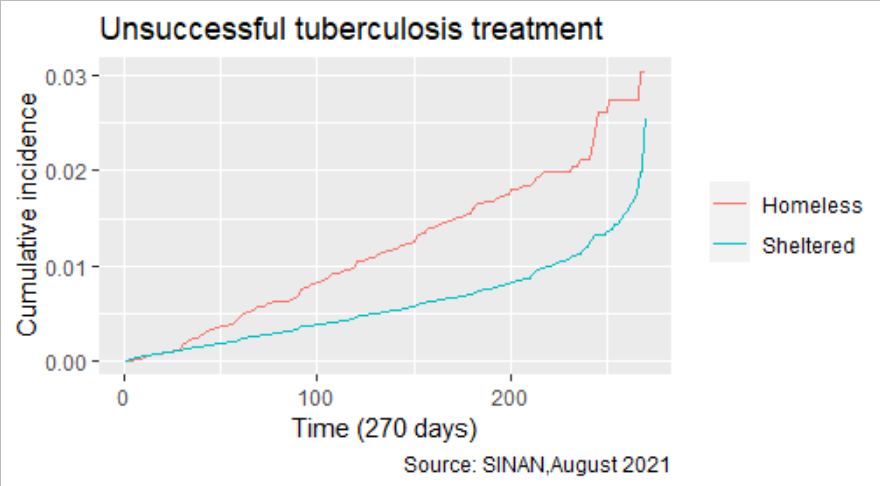

Supplement: S3 Fig — (TIF) [file pntd.0011685.s003.tif]
