# Supplementary material for: Factors associated with unsuccessful tuberculosis treatment among homeless persons in Brazil: A retrospective cohort study from 2015 to 2020
Source: PLoS Negl Trop Dis. 2023 Oct 20;17(10):e0011685. doi: 10.1371/journal.pntd.0011685 (PMC10619819; doi:10.1371/journal.pntd.0011685)
Supplement: S1 Table — (DOCX) [file pntd.0011685.s006.docx]

**S1 Table.** Sociodemographic and clinical characteristics of Brazilian tuberculosis patients during 180 days of treatment (n=94,639).

|  | Sheltered population | | Homeless population | | P -value  (α =0.05) |
| --- | --- | --- | --- | --- | --- |
|  | n= 89,987 | 95.08% | n= 4,652 | 4.92% |  |
|  |  |  |  |  |  |
| **Sex** |  | |  | | **<0.001*** |
| Female | 24,964 | 27.74 | 819 | 17.61 |  |
| Male | 65,015 | 72.25 | 3,833 | 82.39 |  |
| Missing | 8 | 0.01 | - | - |  |
| **Skin color** |  | |  |  | **<0.001** |
| White | 24,219 | 26.93 | 1,050 | 22.57 |  |
| Black or mixed ethnicity | 52,975 | 58.86 | 2,811 | 60.43 |  |
| Asian | 513 | 0.57 | 20 | 0.43 |  |
| Indigenous | 800 | 0.89 | 20 | 0.43 |  |
| Missing | 11,470 | 12.75 | 751 | 16.14 |  |
| **Age group** |  | |  | | **<0.001** |
| 18 - 29 | 26,104 | 29.01 | 856 | 18.40 |  |
| 30 - 39 | 19,084 | 21.21 | 1,542 | 33.15 |  |
| 40 - 49 | 15,127 | 16.81 | 1,248 | 26.83 |  |
| 50 - 59 | 13,081 | 14.54 | 709 | 15.24 |  |
| 60 - 69 | 9,072 | 10.08 | 232 | 4.99 |  |
| 70 - 90 | 7,519 | 8.35 | 65 | 1.39 |  |
| **Levels of education** |  | |  |  | **<0.001** |
| Bachelor or higher levels | 1,445 | 1.61 | 40 | 0.86 |  |
| Tertiary education | 5,140 | 5.71 | 234 | 5.03 |  |
| Secondary education | 6,465 | 7.18 | 453 | 9.74 |  |
| Illiterate | 393 | 0.43 | 29 | 0.62 |  |
| Missing | 76,544 | 85.06 | 3,896 | 83.75 |  |
|  |  |  |  |  |  |
| **Beneficiary of government cash transfer program** |  |  |  |  | **<0.001** |
| No | 53,049 | 58.95 | 2,262 | 48.62 |  |
| Yes | 4,830 | 5.37 | 205 | 4.41 |  |
| Missing | 32,108 | 35.68 | 2,185 | 46.97 |  |
| **Clinical features of tuberculosis** |  | |  |  | **<0.001** |
| Extra-pulmonary tuberculosis | 12,064 | 13.41 | 231 | 4.97 |  |
| Pulmonary tuberculosis | 77,923 | 86.59 | 4,421 | 95.03 |  |
| **Region of Brazil** |  |  |  |  | **<0.001** |
| Southeast | 37,047 | 41.17 | 2,423 | 52.09 |  |
| North | 11,746 | 13.05 | 260 | 5.59 |  |
| Northeast | 24,498 | 27.22 | 844 | 18.14 |  |
| Central-west | 4,944 | 5.50 | 267 | 5.74 |  |
| South | 11,752 | 13.06 | 858 | 18.44 |  |
|  |  | |  |  |  |
| **HIV** |  | |  |  | **<0.001** |
| No coinfection | 76,765 | 85.31 | 3,483 | 74.87 |  |
| Coinfection | 12,851 | 14.28 | 1,146 | 24.64 |  |
| Missing | 371 | 0.41 | 23 | 0.49 |  |
| **Alcohol misuse** |  | |  |  | **<0.001** |
| No | 67,092 | 74.56 | 1,810 | 38.91 |  |
| Yes | 17,995 | 19.99 | 2,572 | 55.28 |  |
| Missing | 4,900 | 5.45 | 270 | 5.80 |  |
| **Diabetes** |  | |  |  | **<0.001** |
| No | 77,845 | 86.51 | 4,080 | 87.70 |  |
| Yes | 7,465 | 8.30 | 192 | 4.13 |  |
| Missing | 4,677 | 5.19 | 380 | 8.17 |  |
| **Mental disorder** |  | |  |  | **<0.001** |
| No | 82,823 | 92.04 | 3,946 | 84.82 |  |
| Yes | 2,190 | 2.43 | 298 | 6.41 |  |
| Missing | 4,974 | 5.53 | 408 | 8.77 |  |
| **Tobacco use** |  | |  |  | **<0.001** |
| No | 62,634 | 69.60 | 2,043 | 43.92 |  |
| Yes | 22,230 | 24.70 | 2,246 | 48.28 |  |
| Missing | 5,123 | 5.69 | 363 | 7.80 |  |
| **Illicit drug use** |  | |  |  | **<0.001** |
| No | 71,007 | 78.91 | 1,685 | 36.22 |  |
| Yes | 13,390 | 14.88 | 2,638 | 56.71 |  |
| Missing | 5,590 | 6.21 | 329 | 7.07 |  |
|  |  |  |  |  |  |
| **Directly observed treatment-DOT** |  |  |  |  | **0.11** |
| No | 24,655 | 27.39 | 1,227 | 26.38 |  |
| Yes | 35,388 | 39.33 | 1,898 | 40.79 |  |
| Missing | 29,944 | 33.28 | 1,527 | 32.82 |  |
|  |  |  |  |  |  |
| **Treatment outcomes** |  |  |  |  | **<0.001*** |
| Treatment success | 31,967 | 35.52 | 454 | 9.76 |  |
| Loss to follow-up | 23,544 | 26.16 | 2,467 | 53.03 |  |
| Death | 21,869 | 24.30 | 1,080 | 23.22 |  |
| Treatment failure | 96 | 0.11 | 4 | 0.08 |  |
| Not evaluated | 12,511 | 13.91 | 647 | 13.91 |  |

*Fisher exact test
