# Supplementary material for: Factors associated with unsuccessful tuberculosis treatment among homeless persons in Brazil: A retrospective cohort study from 2015 to 2020
Source: PLoS Negl Trop Dis. 2023 Oct 20;17(10):e0011685. doi: 10.1371/journal.pntd.0011685 (PMC10619819; doi:10.1371/journal.pntd.0011685)
Supplement: S2 Table — (DOCX) [file pntd.0011685.s007.docx]

**S2 Table.** Sociodemographic and clinical characteristics of Brazilian tuberculosis patients during 360 days of treatment (n=308,791).

|  | Sheltered population | | | Homeless population | | | | P -value  (α =0.05) | |
| --- | --- | --- | --- | --- | --- | --- | --- | --- | --- |
|  | n= 300,500 | 97.32% | n= 8,291 | | 2,68% | |  | |  |
|  |  |  |  | |  | |  | |  |
| **Sex** |  | | |  | | | | **<0.001** | |
| Female | 92,431 | 30.76 | 1,403 | | 16.92 | |  | |  |
| Male | 208,051 | 69.23 | 6,888 | | 83.08 | |  | |  |
| Missing | 18 | 0.01 | - | | - | |  | |  |
| **Skin color** |  | | |  | |  | | **<0.001** | |
| White | 87,429 | 29.09 | 1,945 | | 23.46 | |  | |  |
| Black or mixed ethnicity | 168,638 | 56.12 | 4,898 | | 59.08 | |  | |  |
| Asian | 1,753 | 0.58 | 49 | | 0.59 | |  | |  |
| Indigenous | 2,634 | 0.88 | 40 | | 0.48 | |  | |  |
| Missing | 40,046 | 13.33 | 1,359 | | 16.39 | |  | |  |
| **Age group** |  | | |  | | | | **<0.001** | |
| 18 - 29 | 92,564 | 30.80 | 1,460 | | 17.61 | |  | |  |
| 30 - 39 | 65,178 | 21.69 | 2,617 | | 31.57 | |  | |  |
| 40 - 49 | 50,953 | 19.96 | 2,247 | | 27.10 | |  | |  |
| 50 - 59 | 44,404 | 14.77 | 1,379 | | 16.63 | |  | |  |
| 60 - 69 | 28,297 | 9.42 | 465 | | 5.61 | |  | |  |
| 70 - 90 | 19,104 | 6.38 | 123 | | 1.48 | |  | |  |
| **Levels of education** |  | | |  | |  | | **<0.001** | |
| Bachelor or higher levels | 8,631 | 2.87 | 84 | | 1.01 | |  | |  |
| Tertiary education | 25,193 | 8.38 | 518 | | 6.25 | |  | |  |
| Secondary education | 27,313 | 9.09 | 950 | | 11.46 | |  | |  |
| Illiterate | 1,529 | 0.51 | 59 | | 0.71 | |  | |  |
| Missing | 237,834 | 79.15 | 6,680 | | 80.57 | |  | |  |
|  |  |  |  | |  | |  | |  |
| **Beneficiary of government cash transfer program** |  |  |  | |  | | **<0.001** | |  |
| No | 167,848 | 55.86 | 3,903 | | 47.08 | |  | |  |
| Yes | 15,730 | 5.23 | 410 | | 4.94 | |  | |  |
| Missing | 116,922 | 38.91 | 3,978 | | 47.98 | |  | |  |
| **Clinical features of tuberculosis** |  | | |  | |  | | **<0.001** | |
| Extra-pulmonary tuberculosis | 39,143 | 13.03 | 397 | | 4.79 | |  | |  |
| Pulmonary tuberculosis | 261,357 | 86.97 | 7,894 | | 95.21 | |  | |  |
| **Region of Brazil** |  |  |  | |  | | **<0.001** | |  |
| Southeast | 144,808 | 48.19 | 4,560 | | 55.00 | |  | |  |
| North | 35,099 | 11.68 | 488 | | 5.88 | |  | |  |
| Northeast | 70,591 | 23.49 | 1,381 | | 16.66 | |  | |  |
| Central-west | 14,198 | 4.72 | 433 | | 5.22 | |  | |  |
| South | 35,804 | 11.92 | 1,429 | | 17.24 | |  | |  |
|  |  | | |  | |  | |  | |
| **HIV** |  | | |  | |  | | **<0.001** | |
| No coinfection | 272,204 | 90.59 | 6,555 | | 79.06 | |  | |  |
| Coinfection | 27,291 | 9.08 | 1,700 | | 20.50 | |  | |  |
| Missing | 1,005 | 0.33 | 36 | | 0.44 | |  | |  |
| **Alcohol misuse** |  | | |  | |  | | **<0.001** | |
| No | 238,850 | 79.48 | 3,385 | | 40.83 | |  | |  |
| Yes | 49,489 | 16.47 | 4,506 | | 54.35 | |  | |  |
| Missing | 12,161 | 4.05 | 400 | | 4.82 | |  | |  |
| **Diabetes** |  | | |  | |  | | **<0.001** | |
| No | 262,940 | 87.50 | 7,373 | | 88.93 | |  | |  |
| Yes | 25,480 | 8.48 | 333 | | 4.02 | |  | |  |
| Missing | 12,080 | 4.02 | 585 | | 7.06 | |  | |  |
| **Mental disorder** |  | | |  | |  | | **<0.001** | |
| No | 281,477 | 93.67 | 7,159 | | 86.35 | |  | |  |
| Yes | 6,456 | 2.15 | 510 | | 6.15 | |  | |  |
| Missing | 12,567 | 4.18 | 622 | | 7.50 | |  | |  |
| **Tobacco use** |  | | |  | |  | | **<0.001** | |
| No | 219,313 | 72.98 | 3,829 | | 46.18 | |  | |  |
| Yes | 68,642 | 22.84 | 3,930 | | 47.40 | |  | |  |
| Missing | 12,545 | 4.18 | 532 | | 6.42 | |  | |  |
| **Illicit drug use** |  | | |  | |  | | **<0.001** | |
| No | 249,282 | 82.95 | 3,402 | | 41.03 | |  | |  |
| Yes | 37,316 | 12.42 | 4,383 | | 52.87 | |  | |  |
| Missing | 13,902 | 4.63 | 506 | | 6.10 | |  | |  |
|  |  |  |  | |  | |  | |  |
| **Directly observed treatment-DOT** |  |  |  | |  | | **0.003** | |  |
| No | 115,555 | 38.45 | 3,067 | | 36.99 | |  | |  |
| Yes | 114,459 | 38.09 | 3,164 | | 38.16 | |  | |  |
| Missing | 70,486 | 23.46 | 2,060 | | 24.85 | |  | |  |
|  |  |  |  | |  | |  | |  |
| **Treatment outcomes** |  |  |  | |  | | **<0.001** | |  |
| Treatment success | 229,998 | 76.54 | 3,517 | | 42.42 | |  | |  |
| Loss to follow-up | 31,517 | 10.49 | 2,858 | | 34.47 | |  | |  |
| Death | 23,165 | 7.71 | 1,125 | | 13.57 | |  | |  |
| Treatment failure | 198 | 0.07 | 6 | | 0.07 | |  | |  |
| Not evaluated | 15,622 | 5.19 | 785 | | 9.47 | |  | |  |
