# Supplementary material for: Factors associated with unsuccessful tuberculosis treatment among homeless persons in Brazil: A retrospective cohort study from 2015 to 2020
Source: PLoS Negl Trop Dis. 2023 Oct 20;17(10):e0011685. doi: 10.1371/journal.pntd.0011685 (PMC10619819; doi:10.1371/journal.pntd.0011685)
Supplement: Attachment — Submitted filename: Response to Reviewers.docx [file pntd.0011685.s008.docx]

**RESPONSE TO REVIEWERS**

**Reviewer 1:**

**Comment 1:** This study tries to compare the TB treatment outcomes between patients who are homeless with those not homeless. The design and the groups included in the study are appropriate.

**Response 1:** Thank you very much for your comments

**Comment 2:** However, the objectives though scientifically valid, need to be stated using language that is clear.

**Response 2:** We thank the authors for this comment. We have now revised our text and provided very clear objectives. Please see the last 5 lines of paragraph 3 of the introduction.

**Paragraph 3:**

Despite the social scenario that Brazilian HP are exposed to, there are paucity of longitudinal studies focusing on TB epidemiology among this group. It is therefore necessary to investigate the social and health determinants that influences TB treatment and outcomes among HP in Brazil. This study therefore evaluated factors associated with unsuccessful TB treatment outcomes among homeless population (HP) compared to those with shelter. The specific objectives of this study were to; (1) explore the relationship between homelessness and tuberculosis (TB) treatment outcomes , (2) identify factors associated with unsuccessful TB treatment among HP, and (3) determine the significant variables that contribute to undesirable treatment outcomes

**Comment 3:** Sample size has not been calculated for this study. It will be good to calculate and see whether the numbers finally available for complete analysis are enough to draw valid conclusions.

**Response 3:** Thank you very much for your comments. We confirm that the power of our sample size is adequate. We have used the “pwr” package in R, to test the power of our current sample size using the codes below, where n1 and n2 are the sample size in the two groups, and 2.04 is the hazard ratio;

pwr.t2n.test (n1 = 832, n2 = 31830, d = log(2.04), sig.level = 0.05, power = NULL, alternative = "two.sided")

The results show a power of 1.0, which indicates that the sample size is adequate to determine the effect size with 100% probability, if the effect is true.

```
	  t test power calculation

             n1 = 832
              n2 = 31830
              d = 0.7129498
     	   sig.level = 0.05
          power = 1
   	 alternative = two.sided
```

**Comment 4:** It is understood that the database represents all TB patients in the country. But there are large numbers with missing values. Therefore, it will be good to check the characteristics of those omitted from the analysis with those finally included. It is possible that the patients who got included were not representative of all TB patients.

**Response 4:** We thank the reviewer for this invaluable comment. The point raised about the missing values is very correct, and caused by lack of synergy among the varying sources that feeds the SINAN website. We consider this as a limitation and have included a section in the later end of the manuscript. We have expanded the section of limitations significantly, and described the limitations in details

**Please see below:** Lines 370-398

This study has some limitations. Foremost is the large number of missing values which led to the omission of some records in our final models. We believe the characteristics of the records omitted might further enrich our analysis. It is therefore important to invest efforts in ensuring synergies and closing gaps that exist in data reportage among all the varying sources that feeds the SINAN. Secondly, access to safe and nutritious food is an important factor that influences clinical conditions of TB patients, and homeless population are mostly in need of sufficient and nourishing meals [26]. These population are most likely to prioritize acquisition of food and shelter over other health-seeking behavior such as TB treatment [32]. As such, if homeless population have access to these primary necessities, it could better facilitate their willingness to complete TB treatment [33]. However, our models only included covariates in the SINAN database, and the covariate on nutritional status or access to food supply was not captured on the database. Thirdly, the notification end date does not necessarily coincide with the treatment end date, but the date the case was closed on SINAN. Thus, the use of the closing date can lead to interpretations that do not necessarily reflect the duration of treatment. To reduce doubts, we performed the sensitivity analyses to confirm if the results would be the similar over different periods. Fourthly, some TB deaths may be registered at the Mortality Information System (*Sistema de Informação sobre Mortalidade* -SIM), and since we did not link both system (i.e., SINAN and SIM), some of the mortality data might have been underestimated. Finally, since TB is an infectious disease related to poverty [17], and most homeless populations receive lower or no income [6], investigating the impact of cash transfer programs could better highlight the influence of social support policies on TB treatments and outcomes. Unfortunately, we couldn’t explore this association because of the large amounts of missing data in our dataset. However, previous studies by Andrade et al. [29], found a higher proportion of TB treatment were observed among people that received government aids [29]. Since more than 45% of the homeless population in our study are non-beneficiaries of cash-transfer programs, it is therefore important for government to invest in more innovative and inclusive strategies that increases the coverage of financial aids to these vulnerable population.

**Comment 5**: Statistical analysis is appropriate for the stated objectives.

**Response 5:** We thank the reviewer for this invaluable comment.

**Comment 6:** The analysis is appropriately done and results represented in tables. However, the table titles need to be modified in more intelligible English. Figures need to competed and self-explanatory with titles and foot notes.

**Response 6:** We thank the reviewer for this invaluable comment. We have revised our text extensively, including the table and figures (and their title). We have merged several tables together to provide coherence (Table 2 and 3 now Table 2), Table 4 and 5, now Table 3). We have also modified Fig 1 and 2 appropriately

Please see the title of the tables below:

**Table 1: -** Sociodemographic and clinical characteristics of tuberculosis patients in Brazil (n= 284,874; Year: 2015-2020)

**Table 2.** Association between socio-demographic and clinical covariates and the risk of unsuccessful TB treatments among sheltered and homeless populations in Brazil (2015-2020)

**Table 3.** Factors associated with undesirable treatment outcomes among homeless populations in Brazil (n= 830; Year: 2015-2020)

**Comment 7:** The analysis is appropriate, but does not include many variables that can influence the TB treatment outcomes, such as nutritional status and severity of disease. Information on these variables is difficult to obtain from country wide databases. But this can be one reason why alcohol use was found to be protective against adverse outcomes. Missing data is a major issue which has been discussed, but no attempt made to identify or quantify its effect on the effect measures.

**Response 7:** We thank the reviewer for this invaluable comment. The point raised about the lack of other important covariates in the model is very correct. We consider this as a limitation and have included a section in the later end of the manuscript.

**Please see below:** Lines 370-398

This study has some limitations. Foremost is the large number of missing values which led to the omission of some records in our final models. We believe the characteristics of the records omitted might further enrich our analysis. It is therefore important to invest efforts in ensuring synergies and closing gaps that exist in data reportage among all the varying sources that feeds the SINAN. Secondly, access to safe and nutritious food is an important factor that influences clinical conditions of TB patients, and homeless population are mostly in need of sufficient and nourishing meals [26]. These population are most likely to prioritize acquisition of food and shelter over other health-seeking behavior such as TB treatment [32]. As such, if homeless population have access to these primary necessities, it could better facilitate their willingness to complete TB treatment [33]. However, our models only included covariates in the SINAN database, and the covariate on nutritional status or access to food supply was not captured on the database. Thirdly, the notification end date does not necessarily coincide with the treatment end date, but the date the case was closed on SINAN. Thus, the use of the closing date can lead to interpretations that do not necessarily reflect the duration of treatment. To reduce doubts, we performed the sensitivity analyses to confirm if the results would be the similar over different periods. Fourthly, some TB deaths may be registered at the Mortality Information System (*Sistema de Informação sobre Mortalidade* -SIM), and since we did not link both system (i.e., SINAN and SIM), some of the mortality data might have been underestimated. Finally, since TB is an infectious disease related to poverty [17], and most homeless populations receive lower or no income [6], investigating the impact of cash transfer programs could better highlight the influence of social support policies on TB treatments and outcomes. Unfortunately, we couldn’t explore this association because of the large amounts of missing data in our dataset. However, previous studies by Andrade et al. [29], found a higher proportion of TB treatment were observed among people that received government aids [29]. Since more than 45% of the homeless population in our study are non-beneficiaries of cash-transfer programs, it is therefore important for government to invest in more innovative and inclusive strategies that increases the coverage of financial aids to these vulnerable population.

**Comment 8:** Overall, the language needs modification, to be presented in more intelligible fashion.

**Response 8:** We thank the reviewer for this invaluable comment. We have revised our text extensively, and made significant text and grammatical checks. As such, our language and presentation have significantly improved.

**Comment 9:** This is an important study highlighting the failure of TB treatment for the most vulnerable section of the society, the homeless. This section needs to be focused for TB programme to succeed. The study utilizes country wide database, which has both advantages and disadvantages. The representativeness is good, but at the same time, large number of missing values reduces validity of the results.

**Response 9:** We thank the reviewer for this invaluable comment. We have addressed to the best of our abilities the comments raised by the reviewer.

**REVIEWER 2**

**Comment 1:** Line 133: Were transferred to other units, unknown cases and missing values included under unsuccessful treatment?

**Response 1:** Thank you very much for this comment. We apologize for the lack of clarity. Only records of patients who were transferred and missing values were included under unsuccessful treatment.

**Comment 2**: Line 169: Selecting independent variables which were significant for composite unsuccessful outcome and applying to individual categories of outcome will not be accurate. All relevant independent variables must be included in the adjusted analysis for unsuccessful outcomes.

**Response 2:** We thank the reviewer for this comment, and we have provided more clarity between lines 181-185. We didn’t select significant independent variables using p-value cut-offs, rather we included all variables, but excluded variables with missing categories. For example, “Region of Brazil” initially has 5 categories, but after removing the missing values, “NA”, some of the categories were lost (only two was left), hence we excluded this variable from the regression model, irrespective of their significance or relevance to the framework of unsuccessful TB outcome.

**Comment 3:** Table 1: Alcohol misuse- Does this population include those who consume alcohol or those with alcohol abuse/misuse. How was it measured during original data collection? Variable could be renamed as alcohol use rather than misuse.

**Response 3:** Thank you for this comment. We agree with your suggestion to change the variable name to “alcohol use”. The variable "alcohol use" is marked by primary health care workers, and then the answer is entered into the information system. We believe the interpretation on whether alcohol use is abusive or not depends on the interview of the professional with the homeless person.

**Comment 4:** Table 1: Numbers with no alcohol use is wrong.

**Response 4:** We thank the reviewer for this great comment. We have now revised the numbers. Also, we have double-checked all other numbers reported in our tables, and corrected them appropriately. Please see corrections as track changes.

**Comment 5**: Line 207: The term cured is used interchangeably with treatment success which is not appropriate.

**Response 5:** We thank the reviewer for this great comment. We have now revised the word. We changed it to “healed”

**Comment 6**: Line 218: Unsuccessful treatment is mentioned as unconcluded treatment. Unconcluded treatment usually means incomplete treatment. It is not appropriate to use this term in this context.

**Response 6:** We thank the reviewer for this great comment. We have now revised the word, and changes it to unsuccessful treatment.

Comment 7: Line 239: The Hazard ratio mentioned is wrongly interpreted

**Response 7:** We thank the reviewer for this great comment. We have now revised the line and interpreted the hazard ratio as appropriate.

Comment 8: Table 3: The n mentioned here is 830; in flowchart it is mentioned as 832.

**Response 8:** We thank the reviewer for this great comment. In the flowchart, we have a total of 832 records, however while performing the cox-model, two additional records were removed by the software because of very few observations in the “Asian” and “Indigenous” under variable “Race”. This is data truncation. Hence the change from 832, to 830. We have now added a footnote under Table 2 to describe this.

**Comment 9:** The discussion and inferences from the study should be better articulated.

**Response 9:** We thank the reviewer for this great comment. In the flowchart, we have a total of 832 records, however while performing the cox-model, two additional records with failures were removed from the homeless population database hence the change from 832, to 830. We have now added a footnote under Table 2 to describe this. We have also modified the flow chart for clarity.

**Comment 10:** Typographical and grammatical errors are seen throughout the manuscript. The language is unclear, making it difficult to follow. The manuscript should be edited to improve the flow and readability of the text.

**Response 10:** We thank the reviewer for this invaluable comment. We have revised our text extensively, and made significant text and grammatical checks. As such, our language and presentation have significantly improved

**Comment 11:** The authors report the risk factors associated with unfavorable treatment outcomes among homeless population which is one of the neglected study group. It is a good attempt by the author to include data over a period of five years. The appropriateness of statistical analysis performed (Cox regression analysis) and their interpretations require more scrutiny by a biostatistician. The discussion and inferences from the study should be articulated in a better manner. The discussion can be made more succinct. Typographical and grammatical errors are seen throughout the manuscript; grammatical errors in few areas seem to change the interpretation of the text.

**Response 11:** We thank the reviewer for this invaluable comment. We have revised our text extensively, and made significant text and grammatical checks. As such, our language and presentation have significantly improved. We have carefully revised the manuscript, included sub-heads in thee result sections, merged the tables, and succinctly discuss our results. We have also expanded our paragraph on the limitation of the study. We believe this version of the manuscript should be more appealing to some extent to the reviewer. Thanks.
