# Supplementary material for: Factors associated with unsuccessful tuberculosis treatment among homeless persons in Brazil: A retrospective cohort study from 2015 to 2020
Source: PLoS Negl Trop Dis. 2023 Oct 20;17(10):e0011685. doi: 10.1371/journal.pntd.0011685 (PMC10619819; doi:10.1371/journal.pntd.0011685)
Supplement: Attachment — Submitted filename: RESPONSE TO REVIEWERS 25.06.docx [file pntd.0011685.s009.docx]

**RESPONSE TO REVIEWERS**

**REVIEWER 1:**

**C1:** Introduction: There are multiple studies on TB among homeless people, also from Brazil.  How does this add to the available knowledge should be added in the justification for the study. There is a description of the homeless people but, not on the burden of TB among the homeless people, critical appraisal of the evidences available and the existing gaps in knowledge can be added in the introduction.

**RESPONSE 1:** We sincerely appreciate the reviewer for this comment, although we initially added a single line in our previous version emphasizing that this current study employed a longitudinal approach in assessing risk, compare to most other studies that have used a cross-sectional approach. As such we have significantly expanded our lines on this, and also added 3 new references. Please see below excerpt [lines 81-103, from clean copy]

Most of them become homeless because of addiction to excessive alcohol consumption and drug abuse, unemployment and family disagreement [8]. According to the Unified Registry for Social Programs (*CADUNICO, Cadastro Único para Programas Sociais)* there were 160,097 HP that received some social benefit in Brazil in March 2021, with 88.8% classified to live in extreme poverty condition and 67.8% of them are of black or mixed ethnicity [11]. Numerous studies have investigated the higher risk of TB and comorbidities among HP compared to the general population, including studies conducted in Brazil and other countries [5, 7, 12, 13]. However, recent research focusing on the epidemiology of TB among this population has predominantly used a cross-sectional approach to estimate risk [7]. As a result, there is a lack of longitudinal studies that specifically examine the TB epidemiology among high-risk populations. Longitudinal studies are essential as they allow for the analysis of trends and patterns over time, offering a more comprehensive understanding of the factors associated with TB in this group. Such studies can provide valuable insights to support ongoing healthcare interventions targeted at high-risk populations. This study therefore employed a longitudinal approach in assessing the social and health determinants that influences TB treatment and outcomes among HP in Brazil. This study therefore evaluated factors associated with unsuccessful TB treatment outcomes among HP compared to those with shelter. The specific objectives of this study were to:; (1) explore the relationship between homelessness and TB treatment outcome, (2) identify factors associated with unsuccessful TB treatment among homeless and sheltered populations, and (3) determine the factors associated with undesirable treatment outcomes among homeless populations.

C2: Study setting: Some details regarding the TB treatment in Brazil, such as 1) the treatment following alternate day or daily dose regimen and 2) whether DOTS is offered to all TB patients in the country, will be helpful for the readers to understand the context.

**RESPONSE 2:** We sincerely appreciate the reviewer for this comment. We have now improved our lines to capture this very important suggestion. Please see below excerpt [lines 118-126, from clean copy]

In Brazil, TB treatment is conducted according to the guidelines established by the Brazilian Ministry of Health. These treatment services are accessible to all citizens through the public healthcare system known as Sistema Único de Saúde (SUS). The treatment is provided free of charge and follows a daily-dosage regimen over a six-month period. While Directly Observed Therapy (DOT) is recommended, there are variations in the treatment models offered across clinics, with some providing self-administered therapy (SAT). The frequency of supervision during the treatment may also vary, with at least three visits per week in the first two months and twice weekly visits for the remaining four months [18].

**C3:** Exclusion criteria: The statement is not clear as to what are the stages being referred to.

**RESPONSE 3:** We absolutely agree with the reviewer that the exclusion criteria was not clear. We have thus revised this section appropriately. Please see below excerpt [lines 130-136, from clean copy]

The inclusion criteria for this study were patients with: (1) new cases of TB, and (2) between age 18 to 90 years. Exclusion criteria includes: (1) TB patients without a clear definition of homelessness or been sheltered, (2) Patients with unknown previous TB treatment history (3) previously treated patients with relapse, (4) patients who were transferred, (5) patients treated after loss to follow-up, (6) post-death cases, (7) patients with drug resistant TB (DR-TB), (8) patients with primary abandonment, and (9) patients who changed therapy

**C4:** Outcome variable: 'Loss to follow-up' (LTFU) and 'not evaluated' seem to be similar for research purpose as the treatment outcome could not have been assessed for either category. Were these two groups combined in the analysis?

**RESPONSE 4:** We thank the authors for the comment. We have revised the section to clarify our definitions for this category. Also, we didn’t combine the two groups in the analysis to allow us describe the demography and better capture the effect of socio-demographic and clinical covariates on LTFU. Please see our improved lines below excerpt [lines 140-153, from clean copy]

Based on the literature [19-21], we defined unsuccessful treatment as falling into one of the following categories: (1) treatment failure, (2) death, (3) loss to follow-up, or (4) not evaluated. The "Treatment failure" category refers to patients who tested positive for sputum smear or culture at the 5th month of treatment or later. The "Death" category includes patients who died due to TB or other causes. The "Loss to follow-up" category pertains to TB patients who had taken medications for 30 days or more but then interrupted treatment for one month or longer. The "Not evaluated" category describes cases where TB patients were either transferred to other healthcare units or their records are unknown, incomplete, or have missing information. On the other hand, the successful treatment category refers to patients who are considered cured if they have two negative smears, one during any month of treatment and the other at the 5th or 6th month of therapy, or if they completed treatment without any signs of failure and were discharged based on clinical and radiological examinations [1,2,19].

**C5:** Covariates: Severity of alcohol and tobacco use and control status of diabetes would have an effect on the treatment outcomes. Dichotomisation of these variables reduces their influence on the outcomes.

**RESPONSE 5:** We appreciate the reviewers for this comment and have captured this as one of the limitations of the study. Please see below our improved lines; [lines 423-427, from clean copy]

However, it's important to note that our models considered the covariates available in the SINAN database. These covariates were mostly dichotomized, which could have limited the variability in the data. Furthermore, the covariate on nutritional status or access to food supply was not captured on the database.

**C6:** Statistical analysis: 1) The covariates included in the model for assessing the association of specific risk factors with LTFU and death, displayed in table 3, are not mentioned.

**RESPONSE 6:** We thank the reviewers for this comment, and we have addressed the methodology section to include the covariates. Also, we would like to emphasize that we also explained in the text describing table 3 how we arrived at the three covariates included in the model that produced table 3. Please see our improved lines below. [lines 200-204, from clean copy]

The crude and adjusted Cox hazards regression (HR) was also performed to identify the factors associated with the unsuccessful treatment among the HP and, also the association between significant predictors (illicit drug use, mental disorder and DOTs) and TB outcome categories.

C7: Lines 173, 174 and 175: Points two and three appear to be same.

**RESPONSE 7**: The objectives are different as the point 2 provides comparison between homeless and sheltered populations, while the point 3 and 4 focused more on homeless populations, with the first objective (point 3), investigating association of homeless and treatment outcomes, and the last objective (point 4), on magnitude of association between significant predictors from objective 3, and outcome category.

**C8:** It is mentioned that sensitivity analysis was performed to see the effect of varying durations of treatment periods. However, the findings of the sensitivity analysis are not described in the result section.

**RESPONSE 8:** We thank the authors for pointing our attention to this important comment. We have addressed this issue earlier, by providing the results of the sensitivity analysis as supplementary file 3 and 4, but we failed to describe them in the main manuscript. However, following your comments, we have revised our text to briefly indicate that findings from the sensitive analysis. Please see below excerpt [lines 163-167, from clean copy]

We performed descriptive sensitivity analyses by comparing treatment periods of 180, 270, and 360 days to evaluate their impact on the outcome results. The findings revealed a p-value below 0.05, indicating that the observed differences are statistically significant and not merely due to chance (see S1 Table and S2 Table for details).

**C9:** Table no 2: How can homelessness be a risk factor for the sheltered group of patients?

**RESPONSE 9:** We are deeply grateful for the eagle eye of the reviewer. This was an oversight. We have now corrected the placings of the values, by assigning Refence to the cells under the shelthered population column and then the HR values to the cells under the homeless population column

**C10:** For tables 2 and 3, only the HR are given for the covariates. The number of outcomes in the various categories of the covariates will add more information.

**RESPONSE 10:** We thank the reviewer for this comment. We have now revised the tables to provide the number as advised.

**C11:** Skin colour as a risk factor for unsuccessful treatment outcome is probably associated with social rather than biological factors. This point could be discussed more.

**RESPONSE 11:** We thank the reviewer for this comment. We have thus revised the text accordingly. Please see the extensive discussion lines we added. [lines 314-329, from clean copy]

The association between skin color and unsuccessful treatment outcome in TB is likely influenced by social factors rather than biological ones. Various studies have highlighted the challenges faced by individuals of black or mixed ethnicity in relation to TB. These challenges include inadequate access to appropriate treatment, higher levels of unemployment, poor housing and transportation conditions, and cultural barriers such as health literacy, beliefs, and stigma related to TB within specific populations [24-30]. Black populations from Brazil, have also been reported to have a lower level of education [31], which has a direct impact on their socio-economic status and increases their vulnerability to tuberculosis (TB) and other infectious diseases associated with poverty. It is therefore crucial to recognize the factors that contribute to the social context, leading to disparities in tuberculosis (TB) treatment outcomes among individuals from specific ethnic backgrounds. The understanding of these social determinants can help inform interventions and policies aimed at addressing the root causes of such disparities and promoting equitable TB care for all populations. Optimizing healthcare interventions that protects neglected black populations from infectious diseases like TB, may also have indirect economic benefits [20].

**C12:** Public health implications of the findings of this study vis-a-vis TB control in Brazil is not effectively discussed.

**RESPONSE 12:** We appreciate the detailed attention the reviewer has given to our manuscript. We have thus provided a paragraph in a succinct version, discussing the public health implication of our findings. Please see below.[lines 397-412, from clean copy]

In summary, our study has significant public health implications for TB control in Brazil, by highlighting social factors impacting TB treatment outcomes among neglected populations. Specifically, we found that homelessness, being of black or mixed ethnicity, lack of Directly Observed Therapy (DOT), HIV co-infection, and drug use were associated with higher rates of loss to follow-up and death. These findings underscore the need to address the specific challenges limiting the adherence of homeless populations to TB and HIV diagnosis and care interventions. It is also crucial to implement targeted interventions aimed at mitigating the socio-economic vulnerabilities experienced by individuals of black or mixed ethnicity, including improving access to education, employment opportunities, and housing conditions. Investments that strengthen integration among public institutions that provide support for vulnerable populations, such as the Consultório na rua (CnaR) are also very essential. Employing a multi-sectoral approach, that considers these social determinants of health, and other psychological, financial, and food insecurity issues faced by these populations can improve the retention and adherence of vulnerable populations to TB treatment, ultimately leading to better TB control outcomes, and presumably those of other infectious diseases in Brazil.

**C13:** No comments for the details provided under study limitations and conclusions.

**RESPONSE 13:** We thank the reviewer for the very detailed and constructive comments. We really appreciate

**C14:** The data presentation has been modified. Still, clarity is lacking in the way the tables are presented.

**RESPONSE 14**: We thank the reviewer for the very detailed and constructive comments. We hereby affirm that we have modified the text and tables as required to provide the necessary clarity.

**C15**: Language corrections are needed in multiple places in the manuscript. Public health implications of homelessness on TB control needs to be strengthened.

**RESPONSE 15:** We thank the reviewer for the very detailed and constructive comments. We hereby affirm that we have modified the entire text and make sufficient corrections to the grammars. We have also expanded a bit more on our discussion as suggested.

**REVIEWER 3**

**C1:** The purpose of the study was to evaluate factors associated with unsuccessful TB treatment and identify the risk factors for undesirable TB treatment outcomes among homeless population in Brazil in comparison to sheltered population.
The study background and introduction are clear with appropriate references from literature. The study is appropriately designed and sample size determination is clearly explained for the study groups. Study population, data source and data collection methodology, outcome variables, covariates are mentioned in detail. The study analysis is appropriate for the study aims. No ethical or regulatory concerns were noted.

**RESPONSE 1**: We thank the reviewer for the positive comment on our manuscript.


C2: The study analysis is appropriate. However, few discrepancies were noted in the stated numbers. In Table 1, n=277, 125 for sheltered population but the total counts to 282,125 for the socio-demographic covariate ‘Region of Brazil’’.

RESPONSE 2: We thank the reviewer for the positive comment on our manuscript. We have reviewed this and corrected accordingly. The error was with the first value, which was 137,294, instead of 132,794. The percentages are correct

**C3:** In Table 2, p-value is missing for Homeless population for the variable ‘Black or mixed ethnicity’.

**RESPONSE 3:** We thank the reviewer for the positive comment on our manuscript. We have reviewed this and corrected accordingly.

**C4:** At line 240, p-value for indigenous population is mentioned as <0.001 but is mentioned as 0.001 in Table 2.

**RESPONSE 4:** We thank the reviewer for the positive comment on our manuscript. We have reviewed this and corrected accordingly.

**C5**: At line 260, p-value mentioned for alcohol use among homeless population is mentioned as 0.03 whereas in Table 2, p-value is mentioned as 0.01.

**RESPONSE** 5: We thank the reviewer for the positive comment on our manuscript. We have reviewed this and corrected accordingly.

**C6:** In the S1 Supporting Information, n=89,987 for sheltered population. However, the total counts to 90,057 for the covariate ‘Skin colour’.

**RESPONSE 6:** We thank the reviewer for the positive comment on our manuscript. We have reviewed this and corrected accordingly. The error was with the first value, which was 24299, instead of 24219. The percentages are correct

**C7:** Reference to S3 appendix and S4 appendix needs to be corrected at line 152.

**RESPONSE 7:** We thank the reviewer for the positive comment on our manuscript. We have reviewed this and corrected accordingly.

**C8:** There are minor typographical errors in the revised document in table 3 (drogues), S1 and S2 supporting information (p-valor) etc.

**RESPONSE 8:** We thank the reviewer for the positive comment on our manuscript. We have reviewed this and corrected accordingly.

.
