# Supplementary material for: Factors associated with unsuccessful tuberculosis treatment among homeless persons in Brazil: A retrospective cohort study from 2015 to 2020
Source: PLoS Negl Trop Dis. 2023 Oct 20;17(10):e0011685. doi: 10.1371/journal.pntd.0011685 (PMC10619819; doi:10.1371/journal.pntd.0011685)
Supplement: Attachment — Submitted filename: Response to Reviewers Comments_11.08.docx [file pntd.0011685.s010.docx]

**ROUND 4:**

**Response to Reviewers Comment**

**Reviewer #1:**

C1: The methods section is appropriate. Most of the queries and comments have been answered adequately. Some of the issues that could not be changed have been mentioned in the manuscript as limitations.

Response: We thank the reviewer for the comments, and the efforts expended towards making sure our manuscript meets required standards.

C2: The results match the stated plan of analysis and are as per the objectives of the study. However, the language of presentation of the results needs to be corrected for grammatical errors and also tense at various parts of the result section.

Response: We thank the reviewer for the comments, and we have now revised our manuscript thoroughly using Grammarly to correct all grammatical errors.

C3: In response to one of the comment of the reviewers, the word 'cured' was replaced by 'healed', which is not appropriate as the tubercular lesions often do not heal completely with post-TB lung sequelae being an accepted entity. The term 'successful treatment' as stated in the operational definitions of the methods section can be used consistently.

Response: We thank the reviewer for the comments, and we have replaced this word with successful treatment . Please see line 234

C4: Tables have many empty rows and columns of cells. Merging of cells can be done to make the tables neater.

Response: We thank the reviewer for the comments, and we have cleaned out these tables. We believe once the tables are formatted by the journal during proofing stage, the empty cells would be merged and further cleaned. We have worked through the tables to ensure correctness.

C5: In table no.2, models have been separately done for homeless and sheltered population. How can there be homelessness in the reference category for the sheltered population and a reference category of sheltered population in the model for HP?

Response: We thank the reviewer for the comment. We have corrected this and provided more clarity, by replacing the title of that row with “Homeless population vs Sheltered population”, with the reference category set as “Sheltered Population”

C6: The findings of this study have public health relevance and the conclusions are supported by the data. Limitations have been added as per the suggestions of the reviewers.

Response: We thank the reviewer for the comment.

C7: The language for this paper needs to be improved. There are grammatical errors throughout the manuscript, more in the result section. Table no 2 needs correction as mentioned above for the results.

Response: We thank the reviewer for the comments all through the manuscript. We have significantly improved the text for grammatical correctness, and also adjusted the Table 2 as required.

C8: This study has public health relevance even though there are some major limitations, which are due to the data source and not the design. Writing especially the grammar needs improvement. Tables need correction.

Response: We thank the reviewer for the comments all through the manuscript. We have significantly improved the text for grammatical correctness, and also adjusted the Table 2 as required.

**Reviewer #3:**

C1: In the S1 Supporting information, n=89,987 for sheltered population. The total for the covariate 'skin colour' (89,977) still does not tally. Kindly confirm the correct values.

Response: We thank the reviewer for the comment. We have corrected this. The Asian Category is 24229 instead of 24219.

C2: Also, kindly format Table 2 to fit the contents. The last 2 columns of the table are visible in the word document (Revised manuscript 16.07(track copy)) but has got truncated in the pdf.

Response: We thank the reviewer for the comment. We have adjusted the layout of the page, and we believe once the tables are formatted by the journal during proofing stage, everything should be fine.

C3: No major revisions. All observations have been addressed except comment 6.

Response: We thank the reviewer for the comment. We have adjusted the layout of the page, and we believe once the tables are formatted by the journal during proofing stage, everything should be fine. For the comment 6 on the supplementary error, with the race on complementary files.
