# Supplementary material for: Factors associated with unsuccessful tuberculosis treatment among homeless persons in Brazil: A retrospective cohort study from 2015 to 2020
Source: PLoS Negl Trop Dis. 2023 Oct 20;17(10):e0011685. doi: 10.1371/journal.pntd.0011685 (PMC10619819; doi:10.1371/journal.pntd.0011685)
Supplement: Attachment — Submitted filename: Response to Reviewers comments1309.docx [file pntd.0011685.s011.docx]

**Response to Reviewers comments**

**Reviewer 1:**

C1: All the comments of the reviewers have been addressed in the revised manuscript. There is only a minor point.

In table 2, results from analysis of two multivariable models have been included. The crude and adjusted HR for homelessness is assumed to be from a model developed with the total study population, which should be 32,662 (31,830 sheltered + 832 homeless). However, the number mentioned is n=331,830.

The remaining table are results from two multivariable models done separately for the sheltered (n=31,830) and homeless populations (832). It is leading to some confusion. It will be better if first row of results, which addresses the first objective of exploring the association of homelessness with the unsuccessful treatment outcomes is presented in a separate table. Table 2 can retain the the title and the contents presenting the association of the sociodemographic and clinical covariates with unsuccessful treatment among the sheltered and homeless population separately, the second objective.

R1: We are extremely grateful to the reviewer for this great comment. We absolutely agree to the edits suggested, in fact it has provided more clarity to the tables. We have thus deleted the first row, and made a separate table as suggested. We once again thank the reviewer for the very detailed feedback on our manuscript.
